# Folic acid supplementation in children with sickle cell disease: study protocol for a double-blind randomized cross-over trial

**DOI:** 10.1186/s13063-020-04540-7

**Published:** 2020-06-29

**Authors:** Brock A. Williams, Heather McCartney, Erin Adams, Angela M. Devlin, Joel Singer, Suzanne Vercauteren, John K. Wu, Crystal D. Karakochuk

**Affiliations:** 1 Food, Nutrition, and Health, Faculty of Land and Food Systems, The University of British Columbia, 2205 East Mall, Vancouver, British Columbia V6T 1Z4 Canada; 2 BC Children’s Hospital Research Institute, 950 W 28th Avenue, Vancouver, British Columbia V5Z 4H4 Canada; 3 Department of Pediatrics, Faculty of Medicine, The University of British Columbia, 4480 Oak Street, Vancouver, British Columbia V6H 3V4 Canada; 4 Department of Pharmacy, BC Children’s Hospital, 4480 Oak Street, Vancouver, British Columbia V6H 3V4 Canada; 5 School of Population and Public Health, The University of British Columbia, 2206 East Mall, Vancouver, British Columbia V6T 1Z3 Canada; 6 The Centre for Health Evaluation and Outcome Science, St. Paul’s Hospital, 588 – 1081 Burrard Street, Vancouver, British Columbia V6Z 1Y6 Canada; 7 Department of Pathology and Laboratory Medicine, BC Children’s Hospital, 4480 Oak Street, Vancouver, British Columbia V6H 3V4 Canada

**Keywords:** Sickle cell disease, Pediatrics, Nutrition, Folic acid, Micronutrient supplementation, Randomized control trial

## Abstract

**Background:**

Sickle cell disease (SCD) is a genetic disorder which causes dysfunctional red blood cells (RBC) and is thought to increase requirements for folate, an essential B vitamin, due to increased RBC production and turnover in the disease. High-dose supplementation with 1–5 mg/d folic acid, synthetic folate, has been the standard recommendation for children with SCD. There is concern about whether children with SCD need such high doses of folic acid, following mandatory folic acid fortification of enriched grains in Canada, and advancements in medical therapies which extend the average lifespan of RBCs. In animal and human studies, high folic acid intakes (1 mg/d) have been associated with accelerated growth of some cancers, and the biological effects of circulating unmetabolized folic acid (UMFA), which can occur with doses of folic acid ≥ 0.2 mg/d, are not fully understood. The objective of this study is to determine efficacy of, and alterations in folate metabolism from high-dose folic acid in children with SCD during periods of folic acid supplementation versus no supplementation.

**Methods:**

In this double-blind randomized controlled cross-over trial, children with SCD (*n* = 36, aged 2–19 years) will be randomized to either receive 1 mg/d folic acid, the current standard of care, or a placebo for 12 weeks. After a 12-week washout period, treatments will be reversed. Total folate concentrations (serum and RBC), different folate forms (including UMFA), folate-related metabolites, and clinical outcomes will be measured at baseline and after treatment periods. The sum of the values measured in the two periods will be calculated for each subject and compared across the two sequence groups by means of a test for independent samples for the primary (RBC folate concentrations) and secondary (UMFA) outcomes. Dietary intake will be measured at the beginning of each study period.

**Discussion:**

As the first rigorously designed clinical trial in children with SCD, this trial will inform and assess current clinical practice, with the ultimate goal of improving nutritional status of children with SCD.

**Trial registration:**

ClinicalTrials.govNCT04011345. Registered on July 8, 2019

## Administrative information

Note: the numbers in curly brackets in this protocol refer to SPIRIT checklist item numbers. The order of the items has been modified to group similar items (see http://www.equator-network.org/reporting-guidelines/spirit-2013-statement-defining-standard-protocol-items-for-clinical-trials/).

**Table:** 

Title {1}	Folic Acid Supplementation in Children with Sickle Cell Disease: Study Protocol for a Double-Blind Randomized Cross-Over Trial
Trial registration {2a and 2b}.	Clinicaltrials.gov: NCT04011345
Protocol version {3}	Version 5; December 10, 2019.
Funding {4}	Canadian Institutes of Health Research (CIHR) Thrasher Research Fund
Author details {5a}	^1^ Food, Nutrition, and Health, Faculty of Land and Food Systems, The University of British Columbia, 2205 East Mall, Vancouver, British Columbia, Canada, V6T 1Z4 ^2^ BC Children’s Hospital Research Institute, 950 W 28th Avenue, Vancouver, British Columbia, Canada, V5Z 4H4 ^3^ Department of Pediatrics, Faculty of Medicine, The University of British Columbia, 4480 Oak Street, Vancouver, British Columbia, Canada, V6H 3V4 ^4^ Department of Pharmacy, BC Children’s Hospital, 4480 Oak Street, Vancouver, British Columbia, Canada, V6H 3V4 ^5^ School of Population and Public Health, The University of British Columbia, 2206 East Mall, Vancouver, British Columbia, Canada, V6T 1Z3 ^6^ The Centre for Health Evaluation and Outcome Science, St. Paul’s Hospital, 588 – 1081 Burrard Street, Vancouver, British Columbia, Canada, V6Z 1Y6 ^7^Department of Pathology and Laboratory Medicine, BC Children’s Hospital, 4480 Oak Street, Vancouver, British Columbia, Canada, V6H 3V4
Name and contact information for the trial sponsor {5b}	Canadian Institutes of Health Research (CIHR) 160 Elgin Street, 10th FloorOttawa ON K1A 0W9Canada +1-613-941-2672
Role of sponsor {5c}	The study sponsor and funders have no role in the study design, collection, management, analysis and interpretation of data, writing of the report or publication decisions.

## Introduction

### Background and rationale {6a}

Sickle cell disease (SCD) is a rare, inherited hemoglobinopathy that affects over 5000 Canadians, including 3000 children and youth [1]. In SCD, variants in the β-globin gene produce an abnormal sickle-hemoglobin molecule (HbS), which forms a rigid sickle shape upon release of oxygen due to polymerization [2, 3]. While the severity of disease can vary among homozygous and heterozygous genotypes, hallmarks of the disease include hemolytic anemia, vaso-occlusion, stroke, ischemic tissue damage, organ failure, and severe pain in the back, extremities, thorax, abdomen, and central nervous system [4–6]. Chronic hemolytic anemia and increased red blood cell (RBC) production and turnover in SCD are thought to increase the requirements of folate, a water-soluble family of compounds that are essential for erythropoiesis [7, 8]. Naturally occurring folate is commonly found in foods such as leafy green vegetables, oranges, beans, and legumes [9], while dietary supplements and folate-fortified foods commonly contain folic acid (FA), the synthetic oxidized version of folate, due to its stability and higher bioavailability [10].

The Canadian Haemoglobinopathy Association recommends supplementation of 1–5 mg per day (mg/d) of FA in individuals with SCD in Canada [1], largely based on studies conducted between 1975 to 2001 that showed individuals with SCD had low blood serum or RBC folate concentrations [11–13]. The lowest recommended dose of 1 mg/d FA, however, provides 6 times more folate than is recommended for healthy children aged 1–3 years [14].

The biological effects of FA are not fully understood. In order to be biologically active, FA is reduced to dihydrofolate by dihydrofolate reductase (DHFR). However, the capacity for DHFR to metabolize large amounts of FA may be limited [15]. Recent studies suggest that the maximal capacity for this process is much lower than was previously thought, so even intakes of FA as low as 0.2 mg will leave an appreciable amount of unmetabolized folic acid (UMFA) to be taken up into circulation [15, 16].

There is limited evidence that FA supplementation improves hematological or clinical outcomes in individuals with SCD [7]. Effectiveness of FA supplementation was largely investigated in the 1970–1980s and presented inconclusive evidence [13, 17–20]. A 1983 trial among 117 Jamaican children with SCD aged 6 months to 4 years, the only trial eligible for inclusion in the Cochrane Review of FA supplementation in individuals with SCD, examined the effect of 5 mg/d FA versus a placebo [21]. Although authors observed an increase in serum folate levels (concentrations below 11 nmol/L were evident in 15% (*n* = 6/39) of children in the placebo group versus none of the folic acid group, while concentrations above 40 nmol/L were evident in 81% (*n* = 33/41) of the folic acid group versus 15% in the placebo (*n* = 15/39) group) after 1 year in those treated with FA, no effect on hemoglobin, growth parameters, or other clinical events was observed [21]. The quality of the evidence, however, was low due to the high risk of bias in random sequence generation and incomplete data, making it difficult to draw conclusions from this trial [7].

There is mounting concern about whether Canadian children with SCD require high doses of FA, especially given the mandatory FA fortification of refined grains, specifically white flour, enriched pasta, and enriched corn meal, in Canada since 1998. It is estimated that Canadian children consume, on average, 331 ± 123 μg (*n* = 2193; children aged 1–3 years) to 699 ± 225 μg (*n* = 2397; adolescent males aged 14–18 years) per day of dietary folate equivalents (from both naturally occurring folate and folic acid) [22]. With intake levels of this magnitude, the majority of Canadian children are predicted to meet the recommended dietary allowance for folate from food sources alone. Furthermore, the Canadian Health Measures Survey in 2011 showed that less than 1% of Canadians (*n* = 5248, 6–79 years) had folate deficiency (RBC folate < 305 nmol/L) and 40% had high RBC folate concentrations (> 1360 nmol/L) [23]. In Canadian children with SCD, a recent Canadian study (*n* = 87, age 2–17 years) reported that there was no evidence of serum folate deficiency among individuals supplemented with 1 mg/d FA [24].

Advancements in medical therapy, specifically the use of hydroxyurea, a medication which extends the average lifespan of RBCs from a mean of 4.1 ± 2.71 days to 21.3 ± 6.44 days through the promotion of fetal hemoglobin concentrations [25, 26], may also decrease folate requirements because of reduced RBC turnover. A 2017 study of US children with SCD (*n* = 72), the majority (96%) of which were prescribed hydroxyurea, reported that discontinuation of 1 mg/d FA for ~ 80 days did not change RBC folate concentrations and mean RBC folate concentrations remained high (4 times higher than the suggested World Health Organization deficiency cutoff of 340 nmol/L [16]) after the discontinuation [27].

There is growing concern that higher FA intakes may lead to adverse health outcomes, as intakes of 1 mg/d FA have been associated with acceleration of mammary tumors in rats [28], and colorectal [29] and prostate [30] cancer in adults. There is also evidence that excess FA intakes may have adverse effects on metabolic health during development [31, 32] suggesting young children undergoing periods of rapid growth and development are especially vulnerable. The association of adverse health outcomes with high FA intake remains controversial, but debate has limited some countries from adopting mandatory nationwide FA fortification programs due to these concerns [33].

As no well-designed randomized controlled trial of FA supplementation on children with SCD has been conducted to date, the aim of this study is to determine if high-dose FA supplementation is efficacious, safe, and warranted in children with SCD.

### Objectives {7}

The primary objective is to assess the efficacy of high-dose FA supplementation by measuring the effect of 12 weeks of 1 mg/d FA on RBC folate concentrations, serum folate concentrations, and clinical outcomes (occurrence of megaloblastic anemia and acute pain crises) in children with SCD, compared to placebo.

The secondary objective is to assess alterations in folate metabolism from high-dose FA supplementation by measuring the effect of 12 weeks of 1 mg/d FA on plasma UMFA concentrations, and folate-related metabolite concentrations (plasma *S*-adenosylmethionine, *S*-adenosylhomocysteine, total homocysteine, vitamin B_12_, and methylmalonic acid) in children with SCD, compared to placebo.

We hypothesize that (1) none of the children will present with folate deficiency (RBC folate < 340 nmol/L) during the study period, (2) mean RBC folate concentrations will be similar following 12 weeks of FA or placebo supplementation, showing no benefit of FA supplementation, and (3) plasma UMFA concentrations will be higher following 12 weeks of FA supplementation compared to levels following the placebo supplementation.

### Trial design {8}

This is a double-blind randomized controlled cross-over trial consisting of a 12 ± 1-week intervention period (treatment of 1 mg/d FA or placebo), followed by a 12 ± 1-week wash-out period and a reversal of treatments. The 12-week duration of the intervention and washout periods is based on the estimation that steady-state conditions of RBCs in children with SCD receiving hydroxyurea is reached before 80 days [27]. Approximately 60% of the children with SCD at BC Children’s Hospital are prescribed hydroxyurea [34]. The 12-week washout period will minimize the risk of any carryover effect of the first treatment.

The distinguishing advantage of a cross-over trial from a conventional parallel-arm trial is that each child will serve as his/her own control, thus reducing the bias of confounding variables (e.g., age, sex, dietary intake) [35]. This design will also allow us higher power (lower sample size requirement) to determine a statistically significant treatment effect [35].

A non-inferiority framework will be used to determine whether placebo is inferior or non-inferior to FA, the current standard, for the maintenance of red blood cell folate concentrations.

## Methods: participants, interventions, and outcomes

### Study setting {9}

This trial will occur at BC Children’s Hospital (Canada), an affiliate academic hospital of the University of British Columbia (Canada).

### Eligibility criteria {10}

Individuals will be eligible for participation if (1) they have a confirmed diagnosis of SCD, (2) they are aged 2–19 years and attend BC Children’s Hospital for medical care, and (3) they have received routine daily supplementation of FA for the prior 12 weeks.

Individuals will be excluded from participation if (1) they have received a blood transfusion in the previous 12 weeks, (2) they are allergic to any components of the supplements (cellulose, methylcellulose, magnesium stearate, and/or titanium dioxide), (3) they have presented with megaloblastic anemia in the previous 12 weeks, (4) they have current pulmonary, renal, and/or cardiac complications (severe or recurrent acute chest syndrome), (5) they routinely take medications known to interfere with B vitamin metabolism (chloramphenicol, methotrexate, metformin, sulfasalazine, phenobarbital, primidone, triamterene, barbiturates), (6) they are currently pregnant, planning to become pregnant in the next 9 months, or currently breastfeeding, (7) they have participated in a clinical research trial in the previous 30 days, (8) they have donated blood in the previous 30 days, or (9) they have an unstable medical condition or laboratory results.

### Who will take informed consent? {26a}

Eligible individuals and their parents/legal guardians will be approached by the study coordinator (BAW) with information regarding the study at the out-patient hematology clinic at BC Children’s Hospital. Written informed consent and child or adolescent assent, as applicable, will be obtained.

### Additional consent provisions for collection and use of participant data and biological specimens {26b}

Not applicable

### Interventions

#### Explanation for the choice of comparators {6b}

In this trial design, each participant will receive the current standard of care, 1 mg/d FA, and a placebo comparator for 12 ± 1 weeks. As previous studies have illustrated children with SCD prescribed routine prophylactic FA (1 mg/d) continue to have mean red blood cell folate concentrations 4 times higher than deficiency cutoffs after discontinuation of FA supplementation [27], a placebo comparator was chosen given that treatment (supplementation) may be unnecessary in children, and folate concentrations are speculated to be adequate regardless of treatment group.

#### Intervention description {11a}

Supplements are packaged in bottles containing 100 opaque capsules. The tablets, bottles, and labels on the bottle are identical in appearance except for a sticker which identifies the treatment group (A or B). Supplements will be dispensed from the BC Children’s Hospital Pharmacy at the beginning of each intervention period. Supplements will contain 1 mg FA or placebo, to be taken daily in the morning with food, for the 12 ± 1-week intervention periods. Individuals who cannot swallow capsules may open the capsules and add the powdered supplement to food products.

Supplements were manufactured by Natural Factors Nutritional Products Ltd. (Coquitlam, British Columbia, Canada), and a Notice of Authorization for the purposes of this clinical trial was obtained from the Natural and Non-Prescription Health Products Directorate of Health Canada on December 4, 2019.

#### Criteria for discontinuing or modifying allocated interventions {11b}

Premature withdrawal from the study may occur due to use of other medications which are contraindicated (FA-containing supplements), poor compliance with study protocols, participant request, and/or serious adverse events. Participants who are planning a pregnancy, or who have a confirmed pregnancy, during the intervention period will be removed from the clinical trial and will to return to receiving FA supplementation, as prescribed by their physician, according to the current standard of care. All previously collected data prior to withdrawal will be retained for analysis.

#### Strategies to improve adherence to interventions {11c}

Adherence to study interventions will be monitored by the lead research pharmacist (EA) using capsule counts after each 12 ± 1-week study period and corroborated by supplement diaries which document date, time, and supplement consumption with or without food. The supplement diary will serve as a daily reminder to enhance adherence throughout the intervention periods.

#### Relevant concomitant care permitted or prohibited during the trial {11d}

Consumption of other supplements containing FA is contraindicated for the duration of the study period. All other non-FA containing medications or natural health products approved or prescribed by a physician will be allowed for the duration of the study period.

#### Provisions for post-trial care {30}

There will be medical follow-up for study participants after adverse events until symptoms have resolved or returned to baseline.

#### Outcomes {12}

##### Blood collection and processing

A 3-h fasting venous blood sample will be collected at BC Children’s Hospital and processed by the BC Children’s Hospital BioBank at baseline and the start of each 12 ± 1-week time period for the duration of the study period (weeks 0,12, 24, and 36). A 3-h fast is needed to avoid the confounding influence of recent food or synthetic FA intakes on serum folate levels, which has been shown to have a peak response in serum at 80 min followed by subsequent declines in serum concentrations [36], while still detecting the presence of UMFA, which has been shown to be detectable for up to 10 h in fasted FA supplement users [37]. Caregivers and/or children will be advised not to take the supplement/placebo the day of the blood collection.

A total of 10 mL of venous blood will be collected in a 6-mL EDTA tube and 4-mL serum tube (BD Diagnostics). For preparation of whole blood hemolysate, whole blood (0.3 mL) will be removed from the 6 mL EDTA tube and diluted 1/11 by adding 3.0 mL of a 1% ascorbic acid solution and subsequently incubated at 37 °C for 30 min. Diluted whole blood will be aliquoted into three labeled microtubes. The remaining volume of the 6 mL EDTA tube will then be centrifuged at 3000 rpm for 15 min at 4 °C, and plasma and buffy coat will be collected. Plasma will be aliquoted into five labeled microtubes, and buffy coat will be aliquoted into a single labeled microtube.

The 4 mL serum tube will be left at room temperature for ~ 30 min (until clotted), centrifuged at 3000 rpm for 15 min at 4 °C, and serum will be collected. Serum will be aliquoted into three labeled microtubes.

All aliquots will be stored at − 80 °C until further analysis.

#### Outcome measures

##### Primary objective: efficacy of high-dose FA supplementation

The primary outcome of the first study objective is total RBC folate concentrations (nmol/L) measured at baseline and endline of the intervention periods (weeks 0, 12, 24, and 36). RBC folate indicates longer term folate status, e.g., previous 3–4 months in healthy red blood cells, whereas serum folate reflects recent status or dietary intake [38]. Serum folate will therefore be measured as a secondary outcome at baseline and endline of the intervention periods (weeks 0, 12, 24, and 36). Serum and RBC total folate will be assessed using two methods, as globally recommended [39, 40]: serum folate using a microbiological assay [41] and RBC folate using liquid chromatography-mass spectrometry (LC-MS) [42]. RBC folate forms (tetrahydrofolate (THF), 5-methyl-THF, 5-formyl-THF, and 5,10-methenyl-THF) will be measured using LC-MS [42]. Total RBC folate concentrations will be determined by subtracting serum folate from whole blood folate with a correction for hematocrit.

Secondary clinical outcomes, the occurrence of acute pain crises and megaloblastic anemia, will be measured at baseline and endline of the intervention periods (weeks 0, 12, 24, and 36). Complete blood counts (CBC) will be prospectively collected from clinical blood work completed in tandem with research blood work. CBCs will be determined using an automated hematology analyzer. Megaloblastic anemia will be defined as an increase in MCV > 3 fL and a reticulocyte count < 100 × 10^9^/L, and/or unexplained neutropenia (platelets < 100 × 10^9^/L) and thrombocytopenia (neutrophils < 1.5 × 10^9^/L).

Acute pain crises occurrence (defined as sudden onset of throbbing and continuous pain which can occur in one area of the body such as the back, joints, or arms/legs, or can move throughout areas of the body) will be determined using participant self-report and will be quantified (number of events) and qualified (severity determined by self-management at home, management in Emergency Department of a hospital, or management during an in-patient admission to a hospital ward).

##### Secondary objective: alterations in folate metabolism from high-dose FA

The primary outcome of the second study objective is plasma UMFA concentrations (nmol/L) measured at baseline and endline of the intervention periods (weeks 0, 12, 24, and 36). Plasma UMFA concentrations will be measured using LC-MS [42], as UMFA is not incorporated into RBCs [43]. The proportion of children with detectable UMFA > 0.2 nmol/L, and the proportion (%) of UMFA that makes up plasma total folate will be determined.

Secondary outcome measures of folate-related metabolites (*S*-adenosylmethionine, *S*-adenosylhomocysteine, and total homocysteine concentrations) [44, 45] will be measured using LC-MS [46, 47] at baseline and endline of the intervention periods (weeks 0, 12, 24, and 36).

Other outcome measures will consist of B vitamin status (vitamins B_12_ and B_6_) measured at baseline and endline of the intervention periods (weeks 0, 12, 24, and 36), and dietary folate intake measured at baseline of the intervention periods (weeks 0, 12, 24). For the determination of status of vitamin B_12_, plasma methylmalonic acid (MMA), a marker of vitamin B_12_ status [48], will be measured using LC-MS [49, 50], and plasma vitamin B_12_ will be measured using an immunoanalyzer. For the determination of status of vitamin B_6_, pyridoxal-5′-phosphate (nmol/L), a marker of vitamin B_6_, will be measured using HPLC [51].

Dietary folate intake in children will be estimated using three 24-h dietary recalls, with a 5-step multiple pass method [52], completed at the start of each 12 ± 1-week study period. Food models will be used to increase the accuracy of food portion estimates. Total dietary folate equivalents (DFEs) will be calculated, which takes into account the differences in bioavailability of dietary folate and synthetic FA (1 DFE = 1 μg dietary folate or 0.6 μg FA added to food) [53]. As the potential confounding bias of dietary FA intakes in children will be minimized due to the cross-over design of the trial (each child will serve as his/her own control), estimated dietary FA intakes are not planned to be included in any statistical analyses.

Gene variants associated with folate metabolism will also be measured at baseline (week 0). Genomic DNA will be extracted from buffy coat. *MTHFR* (677 C>T, *rs*1801133, and 1298 A>C, *rs*1801131) and *DHFR* (*rs*1643649 and *rs*70991108) variants [54], which are known to influence folate metabolism, will be detected using polymerase chain reaction (PCR) [55, 56].

### Participant timeline {13

#### Sample size {14}

Sample size was estimated using data from the recent Nguyen et al. [27] study in the USA to estimate means and standard deviation for RBC folate from children with SCD who had 1 mg/d FA discontinued. This population is similar to our clinical population in terms of national FA fortification policies and clinical management. A non-inferiority approach was used to determine the sample size needed to assess the efficacy of 1 mg/d FA as compared to the placebo. Assuming a single group standard deviation in RBC folate of 150 ng/mL (340 nmol/L) observed post-FA discontinuation, a difference in means between groups of 30 ng/mL (68 nmol/L) [27], power of 0.8, alpha of 0.05, and a non-inferiority margin of 100 ng/mL (227 nmol/L) determined based on clinical significance, 28 children (14 in each treatment order) are needed to confirm that the RBC folate concentrations are at most 100 ng/mL (227 nmol/L) lower during the placebo period than during the FA supplementation period.

Sample size was rounded up to 36 children (18 in each treatment order) to account for attrition and to be powered to detect clinically meaningful differences in our secondary outcome (UMFA concentrations).

### Recruitment {15}

Potential participants will first be provided a brief introduction to the study via email communication and later approached in-person in an outpatient clinic setting at BC Children’s Hospital by the study coordinator (BAW). Informed consent and child/adolescent assent will be obtained in person.

### Assignment of interventions: allocation

#### Sequence generation {16a}

An independent research assistant from Natural Factors Nutritional Products Ltd. (Coquitlam, British Columbia, Canada) assigned blinding codes (A or B) to the FA and placebo supplements. The allocation sequence to treatment A or B was determined by a statistician unassociated with the study, under the direction of a clinical trialist (JS), via computer-generated random numbers in SAS statistical software, stratified in blocks of four to ensure roughly equal distribution of treatment order allocations during the study duration.

#### Concealment mechanism {16b}

The generated allocation sequence and blinding codes were transferred directly via encrypted email from the clinical trialist (JS) to the lead research pharmacist (EA) dispensing the treatments in order to maintain blinding of allocation.

#### Implementation {16c}

The lead research pharmacist (EA) will assign participants to treatment order based on the allocation sequence following the enrolment of participants by the blinded study coordinator (BAW).

### Assignment of interventions: blinding

#### Who will be blinded {17a}

Clinicians/care providers, study personnel, outcome assessors, data analysts, and participants will be blinded to the allocation sequence. The sequence allocation will be kept confidential by research pharmacy personnel until full data analysis has been completed.

#### Procedure for unblinding if needed {17b}

In an emergency situation, in which the health and safety of a participant is at risk, breaking the code of the double-blinded study may be done at the request of a medical practitioner responsible for the medical management of a participant to (1) to reveal treatment assignment to inform further medical treatment, (2) to provide information about the nature and risks of the particular treatment the participant is or has received, or (3) for the Principal Investigator to advise about the nature and risks associated with a particular treatment. The research pharmacy may make the randomization assignment result available to the Principal Investigator (CDK), for communication to the requesting clinician, upon reasonable request.

### Data collection and management

#### Plans for assessment and collection of outcomes {18a}

**Fig. 1:**
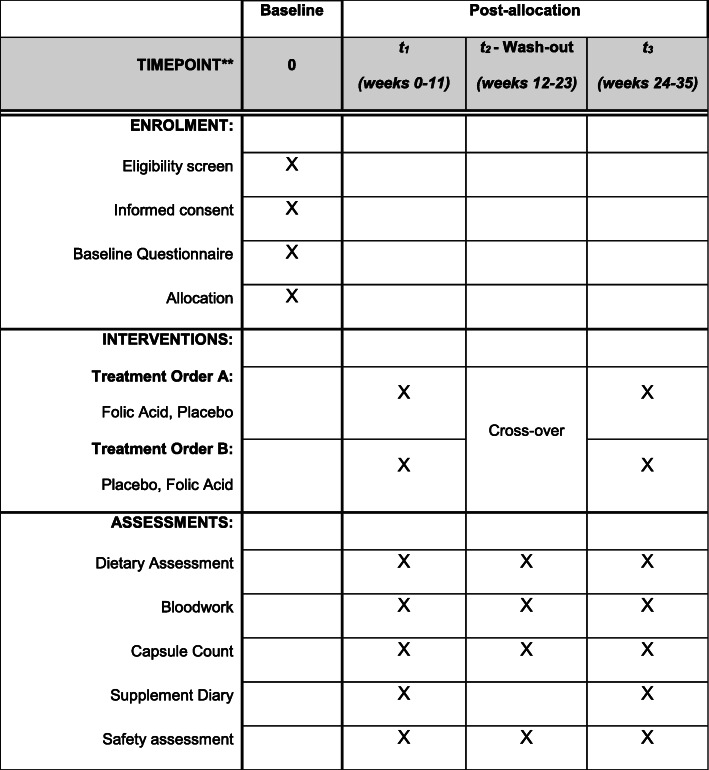
Participant timeline

Data (baseline questionnaires, dietary assessment, clinical outcomes, and adherence) and biochemistry will be prospectively collected throughout the study period according to the participant timeline (Fig. 1). The baseline questionnaire will collect information on disease genotype, region of ancestry, age, current use of supplements and medications (specifically antibiotics, vitamin D, FA, other vitamins and minerals, and hydroxyurea), and history of pain crisis occurrence in the previous 12 weeks.

#### Plans to promote participant retention and complete follow-up {18b}

The provision of incentives ($25 gift card/study visit × 4 study visits) are planned to promote retention of study participants. Study visits are also planned in conjunction with regular clinic appointments to limit the burden of participation and promote complete follow-up.

#### Data management {19}

Study data will be collected and managed using REDCap electronic data capture tools hosted at BC Children’s Hospital Research Institute. Mandatory entry fields, range checks, and double entry in the database will contribute to the promotion of data quality. Quality audits of data will be conducted by research personnel once data collection is complete.

#### Confidentiality {27}

Data with personal identifiers will be stored on an encrypted, password-protected computer in a secure server space with the BC Children’s Hospital Research Institute. A copy of the supplement prescription containing patient identifiers and subject ID, required for dispensing, will be stored under double-lock storage in the hospital research pharmacy. All other data will be de-linked to protect confidentiality. Hard copy data will be stored under double-lock storage for a minimum of 25 years after publication in accordance with Health Canada requirements. Computerized data will be password-protected and stored in the encrypted BC Children’s Hospital Research Institute-maintained RedCap electronic data capture program via hospital network server. Biological samples will be stored in a locked freezer in the Principal Investigator’s (CDK) laboratory at the University of British Columbia.

### Plans for collection, laboratory evaluation, and storage of biological specimens for genetic or molecular analysis in this trial/future use {33}

In the current trial, genomic DNA will be extracted from buffy coat collected at baseline for the detection of *MTHFR* (677 C>T, *rs*1801133, and 1298 A>C, *rs*1801131) and *DHFR* (*rs*1643649 and *rs*70991108) variants. No further genetic or molecular analysis is planned.

### Statistical methods

#### Statistical methods for primary and secondary outcomes {20a}

Intention-to-treat analyses, in which participants will be retained in their original assigned groups, will be performed using Stata 16 (Stata Corp, Texas). Two-sided *p* values less than 0.05 will indicate statistical significance.

First, we will confirm that the washout period was long enough to rule out a carryover effect by conducting a preliminary test: the sum of the values measured in the two periods (for RBC folate concentration) will be calculated for each subject and compared across the two sequence groups by means of a test for independent samples [35]. The 95% CI of the difference in means between treatments will be compared to the non-inferiority margin. If it does not exceed the margin, then we will support the hypothesis that placebo is non-inferior to supplementation [35].

We will repeat the aforementioned analyses for our secondary outcomes (UMFA concentrations). We will also consider plasma UMFA > 0.2 nmol/L as “detectable” levels of UMFA (as reported in previous studies) [57–59] and will calculate the proportion of children with concentrations > 0.2 nmol/L in each group. We will also measure and compare plasma UMFA as a proportion of total blood (% of total blood) across groups. Lastly, we will compare blood folate forms across the groups in proportions of total folate, reduced folate forms, 5-methyl-THF, and UMFA. The “total folate” group will represent the sum of THF, 5-methyl-THF, 5,10-methyl-THF, 5-formyl-THF, and UMFA. The “reduced folate” group will represent the sum of THF, 5-methyl-THF, 5,10-methenyl-THF, and 5-formyl-THF.

#### Interim analyses {21b}

A full review of safety data (occurrence of megaloblastic anemia and acute pain crises, in comparison with baseline measures) will be completed by our independent Data Safety and Monitoring Board (DSMB) after a quarter of the participants (*n* = 9) have completed the first 12 ± 1-week treatment period to ensure the safety of the interventions. Primary outcome measures will not be analyzed at this time.

#### Methods for additional analyses (e.g., subgroup analyses) {20b}

No subgroup or adjusted analyses are planned.

#### Methods in analysis to handle protocol non-adherence and any statistical methods to handle missing data {20c}

An intention-to-treat protocol will be used for data analysis. Multiple imputation will be used to correct for missing data, as appropriate [60].

#### Plans to give access to the full protocol, participant level-data, and statistical code {31c}

The full study protocol is publicly available via ClinicalTrials.gov: NCT04011345. De-identified data and statistical code will be provided to researchers who provide a methodologically sound proposal, following receipt of a signed data access agreement.

### Oversight and monitoring

#### Composition of the coordinating center and trial steering committee {5d}

A multi-disciplinary steering committee was established to guide the research trial from conception to completion. Membership includes the Primary Investigator (CDK), the SCD Clinic Hematologist (JKW), and the SCD Nurse Clinician (HM). The steering committee will also lead knowledge translation planning activities. The data management team will consist of the study coordinator (BAW) and Principal Investigator (CDK).

#### Composition of the data monitoring committee, its role and reporting structure {21a}

An independent DSMB consisting of three independent clinicians that, collectively, have experience in the management of patients with hemoglobinopathies/blood disorders and in the conduct and monitoring of randomized clinical trials has been established.

The purpose of the DSMB meetings is to review the conduct of the trial to date and assess safety of the study intervention. A full review of safety data (occurrence of megaloblastic anemia and acute pain crises, in comparison with baseline measures) will be completed after a quarter of the participants (*n* = 9) have completed the first 12 ± 1-week treatment period. This data will be supplied, in strict confidence, to the DSMB, together with any other analyses that the committee may request. This may include analyses of data from other comparable trials. In the light of this review, the DSMB will advise the TSC (*Trial Steering Committee*) if in its view the study should be discontinued due to safety concerns. As there is evidence that children with SCD maintain adequate RBC folate concentrations following discontinuation of prophylactic FA supplementation, it is expected that the study will continue until planned completion.

#### Adverse event reporting and harms {22}

Independent safety monitoring for the duration of the study will be continuous, with all adverse outcomes being reported during each 12 ± 1-week time period, according to protocol, to our DSMB, research ethics board, and health regulator.

#### Frequency and plans for auditing trial conduct {23}

Trial conduct will follow Good Clinical Practice (GCP) guidelines for the safe and effective undertaking of the clinic trial. The Trial Steering Committee will be in charge of auditing trial conduct, and reviewing trial processes and documents to ensure that research activities comply with the requirements of the protocol on an annual basis. It will review screening and consent documentation to ensure ethical trial conduct.

#### Plans for communicating important protocol amendments to relevant parties (e.g., trial participants, ethical committees) {25}

Any modifications to the protocol which may impact on the conduct of the study and potential benefit of the patient or may affect patient safety, including changes of study objectives, study design, patient population, sample sizes, study procedures, or significant administrative aspects, will be communicated to participants, investigators, research ethics board, regulators, registries, and journals, as applicable.

### Dissemination plans {31a}

Trial results will be disseminated to healthcare professionals, the public, and other relevant groups via open-access peer-reviewed publications and through presentations of findings at both national and international conferences and symposia.

### Trial status

Recruitment began February 2, 2020, and is expected to run until February 1, 2021. The current protocol version (version 5) is dated December 10, 2019.

## Abbreviations

BC: British Columbia
CIHR: Canadian Institutes of Health Research
DSMB: Data and Safety Monitoring Board
FA: Folic acid
RBC: Red blood cell
SAM: *S*-adenosylmethionine
SAH: *S*-adenosylhomocysteine
SCD: Sickle cell disease
TSC: Trial Steering Committee
UBC: University of British Columbia
UMFA: Unmetabolized folic acid
